# Molecular Processes in Stress Urinary Incontinence: A Systematic Review of Human and Animal Studies

**DOI:** 10.3390/ijms23063401

**Published:** 2022-03-21

**Authors:** Wilke M. Post, Joanna Widomska, Hilde Grens, Marieke J. H. Coenen, Frank M. J. Martens, Dick A. W. Janssen, Joanna IntHout, Geert Poelmans, Egbert Oosterwijk, Kirsten B. Kluivers

**Affiliations:** 1 Department of Obstetrics and Gynecology, Radboud University Medical Center, 6525 GA Nijmegen, The Netherlands; wilke.post@radboudumc.nl (W.M.P.); hilde.grens@radboudumc.nl (H.G.); 2 Department of Human Genetics, Radboud University Medical Center, 6525 GA Nijmegen, The Netherlands; joanna.widomska@radboudumc.nl (J.W.); geert.poelmans@radboudumc.nl (G.P.); 3 Radboud Institute of Health Sciences, Department of Human Genetics, Radboud University Medical Center, 6525 GA Nijmegen, The Netherlands; marieke.coenen@radboudumc.nl; 4 Department of Urology, Radboud University Medical Center, 6525 GA Nijmegen, The Netherlands; frank.martens@radboudumc.nl (F.M.J.M.); dick.janssen@radboudumc.nl (D.A.W.J.); egbert.oosterwijk@radboudumc.nl (E.O.); 5 Department of Health Evidence, Radboud University Medical Center, 6525 GA Nijmegen, The Netherlands; joanna.inthout@radboudumc.nl

**Keywords:** stress urinary incontinence, genetic variants, associated genes, associated proteins, gene expression, protein expression, molecular background

## Abstract

Stress urinary incontinence (SUI) is a common and burdensome condition. Because of the large knowledge gap around the molecular processes involved in its pathophysiology, the aim of this review was to provide a systematic overview of genetic variants, gene and protein expression changes related to SUI in human and animal studies. On 5 January 2021, a systematic search was performed in Pubmed, Embase, Web of Science, and the Cochrane library. The screening process and quality assessment were performed in duplicate, using predefined inclusion criteria and different quality assessment tools for human and animal studies respectively. The extracted data were grouped in themes per outcome measure, according to their functions in cellular processes, and synthesized in a narrative review. Finally, 107 studies were included, of which 35 used animal models (rats and mice). Resulting from the most examined processes, the evidence suggests that SUI is associated with altered extracellular matrix metabolism, estrogen receptors, oxidative stress, apoptosis, inflammation, neurodegenerative processes, and muscle cell differentiation and contractility. Due to heterogeneity in the studies (e.g., in examined tissues), the precise contribution of the associated genes and proteins in relation to SUI pathophysiology remained unclear. Future research should focus on possible contributors to these alterations.

## 1. Introduction

Stress urinary incontinence (SUI) is a common and burdensome condition, particularly among parous women [1]. Patients with SUI experience the involuntary leakage of urine synchronous with effort or physical exertion, or on sneezing or coughing [2]. It is the most occurring type of urinary incontinence (UI) in women [3] and has a peak prevalence around the fifth decade of life [4]. Known important environmental risk factors contributing to SUI are increasing age, increased BMI, (multiple) childbirth and other ‘traumatic’ events affecting the endopelvic fascia, and constipation [5,6]. As for menopausal status as a possible risk factor for developing SUI, no consensus has been reached [7,8,9,10]. The current therapeutic strategies consist of pelvic floor physiotherapy, vaginal pessaries, operative repair, or the periurethral injection of bulking agents. These treatments all have different downsides, including general lack of efficacy, poor long term efficacy, or complications due to foreign body reactions [11,12].

SUI is considered a multifactorial trait with bladder neck and urethral incompetence as well as impaired urethral support and levator ani function as contributors [13,14]. In women, SUI often coexists with pelvic organ prolapse (POP), and both are due to lack of support of connective tissues [15,16]. A systematic review of Isali et al. [17] on gene expression differences in SUI that was conducted through September 2017 described 13 genes that were differentially expressed in SUI patients. Those genes are involved in intermediate filament cytoskeleton and extracellular matrix organization. The review did not summarize evidence on animal models and protein expression changes. Several studies indicate a genetic predisposition to SUI [18,19,20] and the molecular pathways involved in SUI have been widely studied but a consensus on the relation to the precise pathophysiology is still lacking. To allow for risk stratification and to develop new preventive or therapeutic strategies more insight into the pathophysiology of SUI is required.

The aim of this systematic review is to synthesize and discuss evidence related to molecular processes in SUI, including genetic variants, gene expression and protein expression differences in both human and animal studies.

## 2. Results

### 2.1. Study Selection

**Figure 1:**
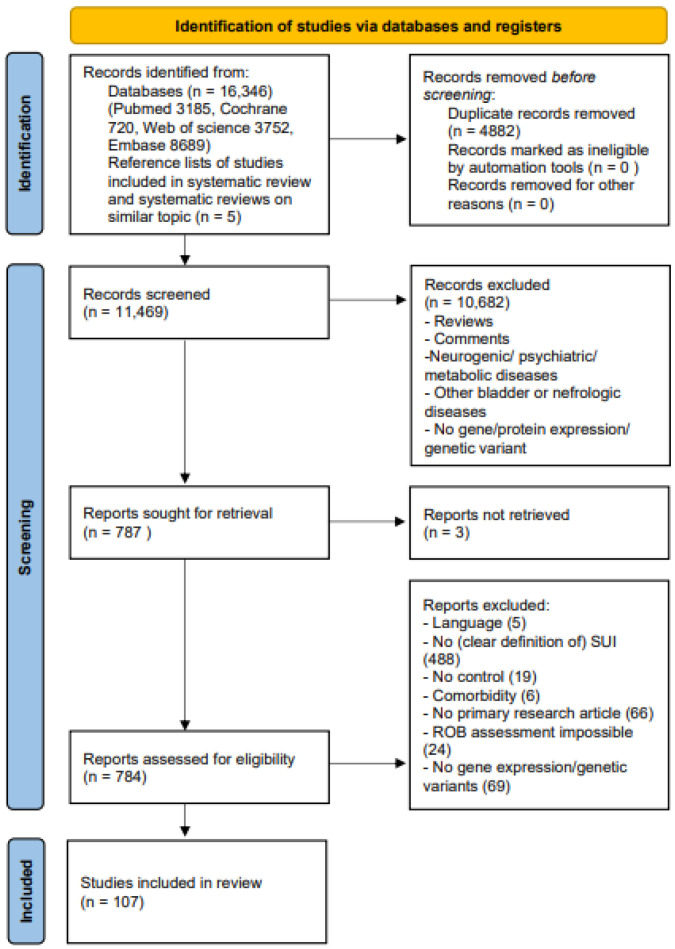
Prisma flow chart [21]. Systematic selection of articles and main reasons for exclusion based on the Prisma 2020 statement. Abbreviations: SUI stress urinary incontinence, ROB risk of bias.

Figure 1 shows the PRISMA flow chart of the review [21]. Of 11,469 retrieved articles, a total of 107 studies (72 human, 35 animal) were included.

### 2.2. Study Characteristics

Supplementary Tables S1 and S2 show the study characteristics of the human and animal studies, respectively. All studies included women and female animals only. Of the human studies, SUI alone was investigated in 52 human studies [22,23,24,25,26,27,28,29,30,31,32,33,34,35,36,37,38,39,40,41,42,43,44,45,46,47,48,49,50,51,52,53,54,55,56,57,58,59,60,61,62,63,64,65,66,67,68,69,70,71,72,73], and SUI with the co-occurrence of POP in 23 studies [62,72,73,74,75,76,77,78,79,80,81,82,83,84,85,86,87,88,89,90,91,92,93], of which three studies investigated both SUI and SUI with POP [62,72,73], All animal studies focused on SUI alone [94,95,96,97,98,99,100,101,102,103,104,105,106,107,108,109,110,111,112,113,114,115,116,117,118,119,120,121,122,123,124,125,126,127,128]. The animal studies used rats (Sprague-Dawley, Lewis, Wistar, and undefined strain) or mice (C57BL/6 strain). The age of the mice and rats used, if reported, ranged from six weeks to six months old. All rats and mice, if mentioned, were virgins or primiparous. SUI induction was mostly performed by vaginal dilatation with various diameters, objects, and durations of dilatation, and with or without bilateral ovariectomy and/or previous spontaneous delivery. Other methods were pudendal nerve crush or transection, electrocauterization, surgically opening of paravesical space, and surgically detaching urethra from the anterior wall. The timeline of the gene or protein expression assessment post-induction of SUI, if reported, ranged from one week until nine months.

Eight human studies discussed genetic variants [48,49,52,62,68,69,84,90], 25 gene expression [25,26,29,32,36,37,38,40,42,44,45,46,47,50,53,61,65,66,67,70,71,73,78,81,87], and 62 protein expression [22,23,24,25,26,27,28,30,31,32,33,34,35,36,37,38,39,40,41,42,43,44,45,46,47,50,51,53,54,55,56,57,58,59,60,61,63,64,65,66,67,70,72,73,74,75,76,77,78,79,80,81,82,83,85,86,87,88,89,91,92,93]. Four animal studies examined genetic variants [107,112,121,122], 11 gene expression [96,101,103,104,111,112,113,115,116,118,120], and 32 protein expression [94,95,96,97,98,99,100,101,102,103,105,106,107,108,109,110,111,112,113,114,116,117,119,120,121,122,123,124,125,126,127,128].

Supplementary Table S3 shows all candidate genes and protein(-related product)s. In total, 179 unique genes/protein(-related product)s were examined (104 by human studies, 96 by animal studies, and 21 by both), and 12 studies focused on the whole genome (*n* = 1) [68], transcriptome (*n* = 5) [36,46,53,71,96] and/or (tissue, serum, or urinary) proteome (*n* = 6) [50,54,63,89,100,107]. One mouse study [107] examined protein expression differences between an SUI model based on *Esr1* knockout and a wild-type control. All other animal studies examined either the gene or protein expression changes after mechanical SUI induction compared to control animals or described the clinical effect of an SUI model based on a specific gene knockout compared to a wild-type animal, without further examining the effect on expression changes.

### 2.3. Risk of Bias of Included Studies

**Figure 2:**
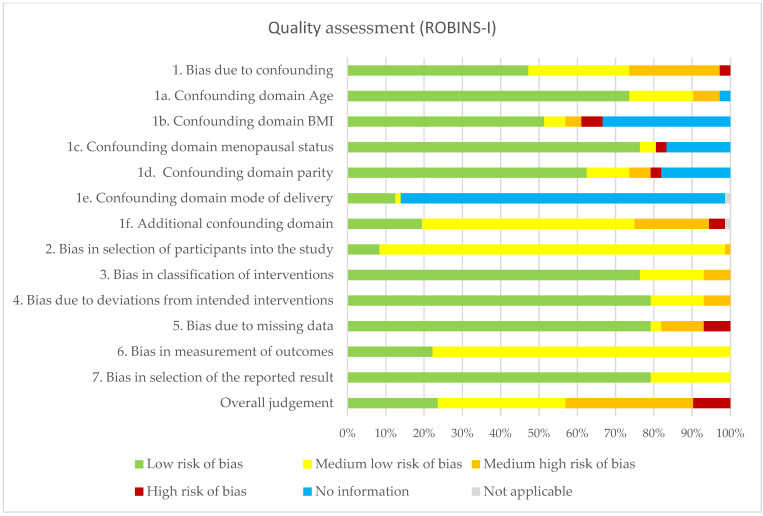
Quality assessment of human studies using the ROBINS-I tool. Abbreviations: BMI body mass index.

**Figure 3:**
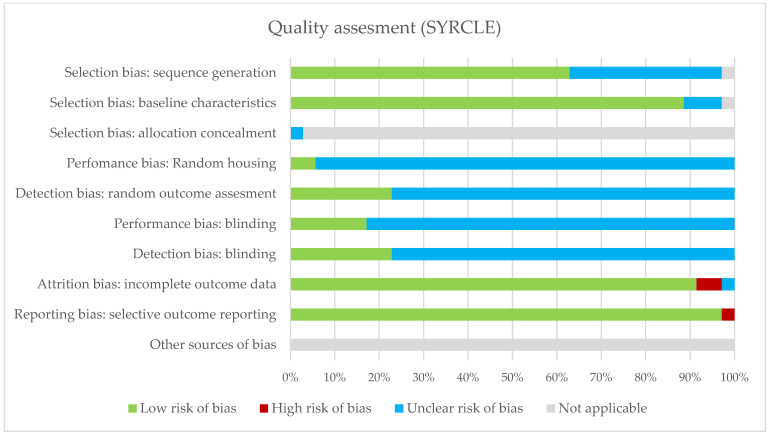
Quality assessment of animal studies using the SYRCLE tool.

The summary of the risk of bias assessment of the human and animal studies is shown in Figure 2 and Figure 3; Supplementary Tables S4 and S5 show a detailed overview per study. Human studies generally scored low risk of bias on the classification of interventions, bias due to deviations from intended interventions, bias due to missing data and bias in the selection of the reported result. Poorer scoring results were assessed in reporting or matching BMI status, mode of delivery and other possibly confounding factors. Overall, 57% scored low or medium low risk of bias in the final judgment of risk of bias. The animal studies generally scored low risk of bias on baseline characteristics, attrition bias and reporting bias. However, many animal studies lacked in reporting housing conditions, detection bias and blinding, resulting in an unclear risk of bias.

### 2.4. Synthesis of the Results

The themes that could be distinguished as overarching for the majority of the results were extracellular matrix (ECM) remodeling, reproduction-related endocrinology, inflammation, oxidative stress, apoptosis, and cell-specific markers and processes. The results were further subdivided between these themes. Table 1 and Table 2 show a summary of analyses of the whole genome, transcriptome and proteome from human and animal studies, respectively. Some of these studies also examined candidate genes; these results are not included in these tables but are discussed below and/or in Supplementary Table S3. Genes and protein(-related products) differentially expressed or associated as indicated by these studies also directed towards these prementioned themes. Genes and/or protein(-related products) not fitting these themes and examined in less than three studies were left out of further discussion.

#### 2.4.1. ECM Remodeling

The most studied topic of research encompasses the ECM. Concerning genetic variants, two [49,84] out of three human studies examining rs1800012 of *COL1A1* in SUI patients and controls showed a significant association between the variant and SUI, the third [69] did not confirm this association. Specific variants in human *FBLN5* [62] and *LOXL1* [52,62] were also associated with SUI. No significant association of specific variants of human *ELN* [62] or *MMP1* [69,90] was shown.

Several differentially expressed genes and proteins in human [36,50,71,89] and animal [96,107] studies examining the whole transcriptome or proteome pointed towards the involvement of the ECM metabolism.

Twenty studies examined ECM-related candidate gene expression [25,26,32,36,37,38,42,45,47,65,67,70,73,78,81,96,111,113,115,116]. Regarding collagen (type 1 and 3) gene expression, both human [25,26,32,45,67,70] and animal [111,113,115] studies show conflicting results irrespective of menopausal status, with the largest human study [32] showing no statistically significant differences in gene expression for both collagen type 1 and 3. Moreover, human studies examining gene expression of the proteoglycans biglycan, decorin, fibromodulin, lumican, and versican show conflicting results of which the majority shows no statistically significant differences [25,26,42,45,73]. Two human studies [38,81] show a lower gene expression of elastase inhibitor alpha-1 antitrypsin, corresponding with another human study [36] showing a higher expression of Elafin (elastase-specific protease inhibitor). Conflicting results were present regarding human *FBN1/2* expression [37,45]. The studies examining matrix metalloproteases (MMPs) gene expression show an increase in human *MMP1* expression [70,78,123] in the SUI group and no statistically significant differences in human [78] and animal [115] *MMP2/Mmp2*, human *MMP3* [78] and animal Mmp13 [96] expression when compared to controls. Conflicting results in human [78] and animal [115] *MMP9/Mmp9* expression were observed, showing no significant change in the human study and increased expression in the animal study comparing SUI subjects against controls. Regarding Tissue Inhibitor of Metalloproteases (TIMP/Timp) studies, *TIMP1* expression in human studies was decreased [70,78], while no significant difference was seen in animal studies of *Timp1* [115], or human [78] or animal [115] studies of *TIMP2/Timp2* [78,115] and human *TIMP3* [78]. As for other proteases, one human study found no significant difference in Calpain-1 expression, however, a higher expression of both Calpain-2 and Calpastatin was seen [47].

**Figure 4:**
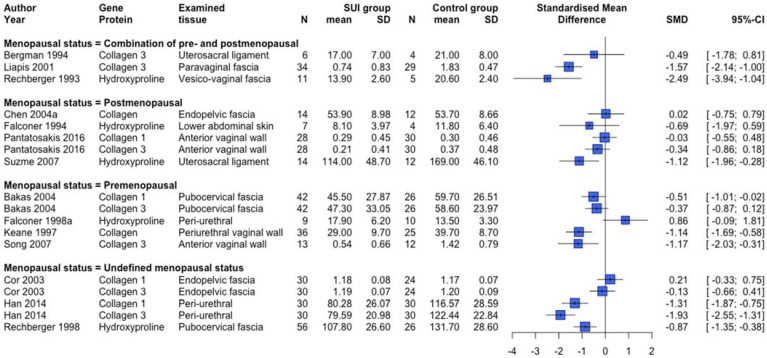
Forest plot of collagen-related protein expression in human studies. This forest plot shows the collagen-related protein expression differences between patients with SUI and controls, subdivided per menopausal status group. Abbreviations: CI confidence interval, N number, SD standard deviation, SMD standardized mean difference, SUI stress urinary incontinence.

**Figure 5:**
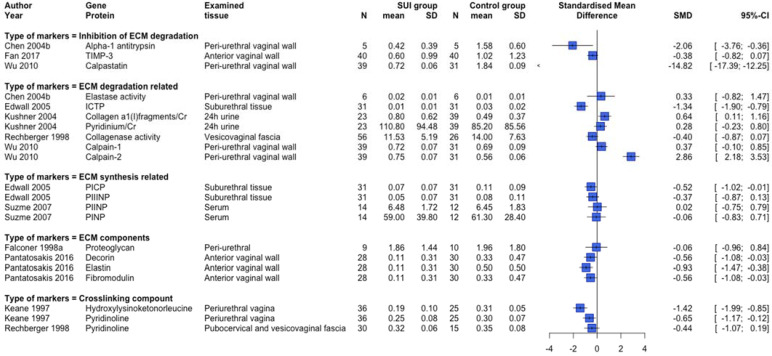
Forest plot of other extracellular matrix related protein expression and biomarker levels in human studies. This forest plot shows other extracellular matrix-related protein expression and biomarker level differences between patients with SUI and controls, subdivided per extracellular matrix metabolism-related theme. Abbreviations: CI confidence interval, N number, SD standard deviation, SMD standardized mean difference, SUI stress urinary incontinence.

ECM-related protein expression and serum or urinary biomarkers were examined in 51 studies [22,23,24,25,26,27,28,30,32,33,34,35,36,37,38,39,41,42,45,47,56,59,65,67,70,73,74,75,76,77,78,81,82,85,93,94,96,97,98,99,108,109,111,112,113,114,119,121,122,126,127]. Figure 4 shows an overview of human [23,24,25,27,30,32,56,73,74,75,77,82,85,93] studies providing quantitative data and examining collagen-related protein expression subdivided per menopausal status group. In line with Figure 4, the majority of human studies not providing quantitative data showed a lower collagen-related protein expression in SUI patients compared to controls [22,26,38,41,45,65,67,70,76,78]. Further, six out of eight animal studies [94,97,111,112,113,114,121,127] showed a lower collagen-related protein expression in the SUI group compared to controls, while the other two [94,114] showed increased expression. Figure 5 displays an overview of the quantitative data provided by human studies examining other ECM-related protein expression and serum/urinary biomarkers. There seems to be a trend towards lower protein expression of ECM macromolecules, crosslinking compounds, and ‘inhibition of degradation’-related proteins. However, no clear difference in ECM synthesis-related protein expression or serum biomarkers was shown. There were, furthermore, some conflicting results regarding degradation-related markers, but the majority of degradation-related protein expression markers and levels of urinary biomarkers show a relatively higher expression in SUI patients versus controls. These results are in line with the studies providing only non-quantitative data [25,26,28,30,36,38,39,41,42,45,65,70,78]. The animal studies examining ECM-related protein expression show conflicting results and no clear trend could be determined (data not shown) [97,98,112,121,126,127].

#### 2.4.2. Reproduction-Related Endocrinology

Expression changes or genetic variants of the reproduction-related endocrinology system were examined by 19 studies [31,39,40,43,48,56,57,58,61,70,74,79,83,99,104,105,107,116,120]. A human study [48] examining polymorphisms of *ESR1* and *AR* showed no statistically significant association. An animal *Esr1* knockout study using an unmentioned strain of mice to create *Esr1* knockout and wild-type mice via the Cre-loxP system, showed a significantly decreased leak-point-pressure (LPP) in the urodynamics of knockout mice when compared to controls, suggesting an association of this gene with SUI [107].

Gene expression changes were examined in three human studies. Only one [61] out of two [40,61] human studies investigating *ESR1* showed a lower gene expression in premenopausal SUI women. No aberrant gene expression changes were shown regarding *ESR1* in studies with postmenopausal women [40,61]. Furthermore, no statistically significant changes were shown in gene expression of *ESR2* [61,70], *AR* [40], and *PR* [40] in studies examining premenopausal women [61], postmenopausal women [61], a combined group [40], or women with undefined menopausal status [70].

**Figure 6:**
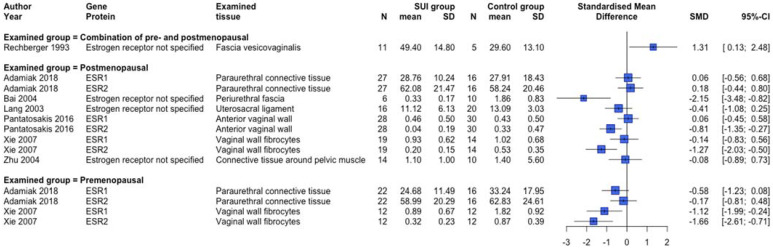
Forest plot of estrogen receptor-related protein expression in human studies. This forest plot shows estrogen receptor-related protein expression differences between patients with SUI and controls, subdivided per menopausal status group. Abbreviations: CI confidence interval, N number, SD standard deviation, SMD standardized mean difference, SUI stress urinary incontinence.

**Figure 7:**
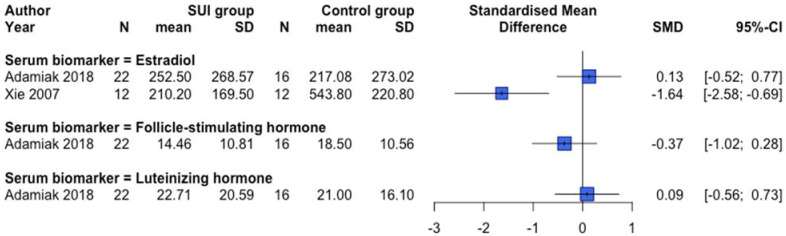
Forest plot of hormonal biomarkers in the serum of premenopausal women. This forest plot shows differences in hormonal biomarkers in the serum between premenopausal patients with SUI and controls, subdivided per hormone. Abbreviations: CI confidence interval, N number, SD standard deviation, SMD standardized mean difference, SUI stress urinary incontinence.

**Figure 8:**
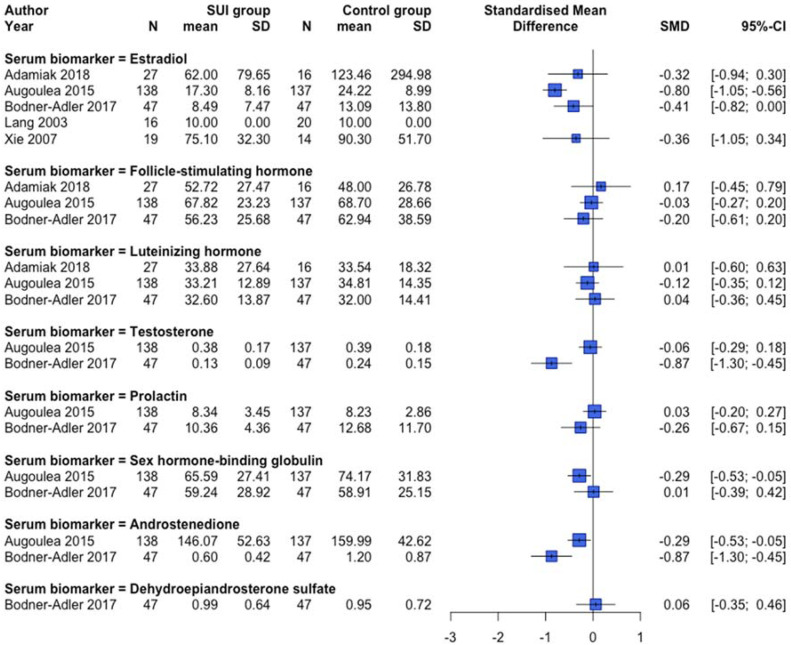
Forest plot of hormonal biomarkers in the serum of postmenopausal women. This forest plot shows differences in hormonal biomarkers in the serum between postmenopausal patients with SUI and controls, subdivided per hormone. Abbreviations: CI confidence interval, N number, SD standard deviation, SMD standardized mean difference, SUI stress urinary incontinence.

Protein expression changes and/or serum biomarkers of the reproduction-related endocrinology system were examined in 12 human [31,39,40,43,56,57,58,61,70,74,79,83] and two animal [105,107] studies. Lower protein expression of ESR1 and ESR2 was observed in premenopausal SUI women (Figure 6), but clear differences dependent on the postmenopausal status or a combined menopausal status group were absent. In another human study not providing quantitative data, no difference in ESR1 protein expression between SUI subjects and controls in both pre and postmenopausal women was found, but conversely, an increased ESR2 protein expression in premenopausal SUI women was detected [40]. An animal study showed increased protein expression of Esr1 among vaginal distended C57BL/6 mice and no changes regarding Esr2 expression [105]. Protein expression of the progesterone receptor was not different between SUI patients compared to controls [31,40,56]. Figure 7 and Figure 8 show an overview of studies examining serum biomarkers in pre and postmenopausal women, respectively. Only serum androstenedione [57,58] (examined in postmenopausal women only) and estradiol in postmenopausal women seem to be lower in SUI patients compared to controls, all other markers show conflicting results or no clear differences. An animal study showed no significant differences in estradiol plasma levels between vaginally dilated C57BL/6 mice versus controls [105].

#### 2.4.3. Oxidative Stress and Apoptosis

Oxidative stress and apoptosis-related genes and proteins were examined in 18 studies [31,64,66,94,103,105,106,112,113,114,115,117,119,122,123,126,127,128]. One animal study showed that *Nfe2l2*-knockout mice had lower LPPs after vaginal dilatation compared to wild-type C57BL/6J mice and this suggests that this antioxidant gene inducer is a potential protective factor in the development of SUI [122].

Several differentially expressed genes and proteins in human [46,63,89] and animal [96,100] studies examining the whole transcriptome or proteome pointed towards the involvement of oxidative stress and/or apoptosis.

Concerning candidate gene expression, a human study showed increased expression of *PERK* and *CHOP* in SUI patients compared to controls [66], suggesting an involvement of endoplasmic reticulum stress and apoptosis in SUI. Two animal studies using Sprague-Dawley rats show conflicting results, namely a lower gene expression of *Casp3* [103], no apparent changes in *Tnfa* [115] and *Tnfr2* [115] expression, and increased expression of *Tnfr1* [115] in the SUI models versus controls. A study using Lewis rat SUI models showed an increased gene expression of *Hif1α* [104].

In line with the gene expression data, PERK protein expression was also increased in human SUI patients [66]. Animal studies showed an increased protein expression of several oxidative stress markers (4-HNE [122], 8-OHdG [122], HO-1 [114], MDA [114,119,122]) in SUI models, except for LDH [103] and p38 [123]. However, multiple animal studies report conflicting results regarding several antioxidative markers [106,113,114,122,127]. Two mice studies [113,119] show a decrease in Nfe2l2 protein expression in SUI models, but a rat study [114] shows no statistically significant change. The majority [64,94,117,128] of the human [64] and animal [94,105,117,128] studies examining neuronal nitric oxide synthase (NOS) expression show a decreased expression in SUI subjects compared to controls. One animal study [117] examining endothelial Nos shows a decrease and two [105,117] out of three [105,106,117] animal studies examining inducible Nos show an increase in SUI models. Animal studies examining Bax and Bcl2 show an increase in Bax(/Bcl2 ratio) [114,122] and both human [66] and animal [122] studies show a decrease in BCL2/Bcl2 protein expression in SUI subjects compared to controls. Several pro-apoptotic markers (Casp3 [122] and Casp9 [122] in mice, Tnfα in rats [126], and CHOP in humans [66]) were increased in SUI subjects, while p53 [31] was decreased in one human study.

#### 2.4.4. Inflammation

Inflammation-related genetic variants, gene and protein expression changes were examined in 21 studies [36,37,54,67,69,85,86,96,98,101,102,104,108,111,112,113,115,123,126,127,128]. One human study showed a non-significant trend towards an association of rs885786 in *SERPINA5* with SUI [69].

Several differentially expressed genes and proteins in human [46,63] and animal [107] studies examining the whole transcriptome or proteome pointed towards the involvement of inflammation. Animal studies examining other gene expression differences show a higher gene expression of *Tnfr1* [115], several cytokines (*Il6* [115], *Ccl7* [101,104], *Ccr1* [104], *Ccr2* [104], *Ccr3* [104]), and cytokine receptor *Cxcr4* [104], and no aberrant changes in *Tnfa* [115], *Tnfr2* [115], and the cytokines *Ccr5* [104], *Cxcl12* [104], *Il1b* [115], and *Il8* [104]. Conflicting results were present in human [37,67] and animal [111,115] studies examining *TGFB1/Tgfb1*, and animal *Smad2* [96,111] and *Smad3* [111] gene expression. One study examining *Acta2* [113] expression in mice showed lower gene expression in the SUI group.

Human studies examining TGFB1 protein expression differences showed lower [67] and no aberrant protein expression differences [37], and a serum measurement study of TGFB1 also showed no aberrant differences [85] in SUI patients compared to controls. (Phosphorylated) Smad2/3/7 were examined in animal studies only, without providing quantitative data, and with conflicting results [96,98,108,111,112,113,127]. Animal studies showed increased protein expression of Tnf-α [126], Il-1 [126], Ccl7 [101] and conflicting results for Tgfb1 [98,111,112,113,127] in SUI models compared to controls. In addition, α-SMA protein expression was examined in animal studies only and was consistently downregulated in SUI subjects compared to controls [98,99,102,113,123,127,128]. A human study showed an increased urinary level of SERPINA5 in SUI patients [54].

#### 2.4.5. Cell-Specific Markers and Processes

**Figure 9:**
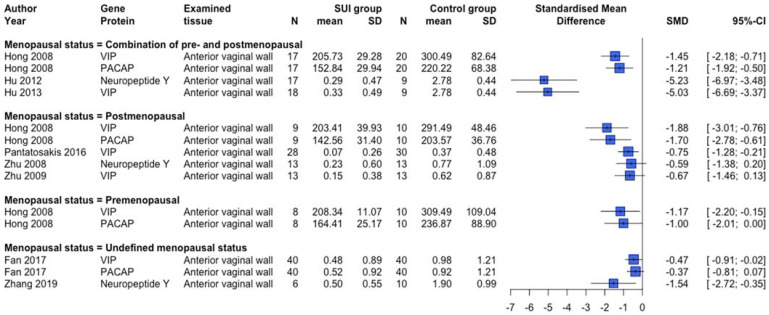
Forest plot of neuron-specific marker-related protein expression in human studies. This forest plot shows differences in the protein expression of neuron-specific markers between patients with SUI and controls, subdivided per menopausal status group. Abbreviations: CI confidence interval, N number, SD standard deviation, SMD standardized mean difference, SUI stress urinary incontinence.

Two human studies examining transcriptomics showed differentially expressed genes and micro-RNAs associated with neurodegeneration [46,53]. In line with these results, an animal transcriptomics study pointed towards neuronal involvement [96]. Generally, a lower protein expression of neuron-specific markers was shown in human SUI patients compared to controls (Figure 9). This is in agreement with human [80] and animal [102,109,124,128] studies not providing quantitative data, except for human neuron specific enolase [80] and Protein S100 [80] that showed no significant changes in protein expression.

Human [50,71,89] and animal [96,100,107] studies study examining transcriptomics and proteomics showed involvement of smooth muscle cell differentiation and/or contraction associated with SUI. Genes and proteins related to muscle cells were examined in animal studies only. Conflicting results were shown for myosin subtypes [106,116,123,128] and desmin [116]. A higher protein expression of the muscle regeneration protein Pax7 was shown in SUI rat models compared to controls [123].

#### 2.4.6. Other

Three animal studies examined Vegf expression [104,116,120] and showed no significant changes in rats induced with SUI compared to controls. However, Vegf protein expression as examined by one rat study [99] was lower in induced-SUI Sprague-Dawley rats compared to controls.

The certainty of evidence assessment according to the GRADE method is shown in Table 3.

## 3. Discussion

To our knowledge, this is the first systematic review of genetic variants, gene and protein expression differences associated with SUI. This review shows that the molecular background of SUI is diverse, which was not unexpected in view of the general consensus that SUI is a multifactorial condition. Nevertheless, through this systematic review, common themes important for SUI were defined at the molecular level.

### 3.1. Principal Findings

SUI is associated with an aberrant ECM metabolism, most likely resulting in a lower gene and protein expression of ECM macromolecules (collagen, elastin, proteoglycans) and crosslinking compounds, and altered degradation, when compared to controls. ECM metabolism is a complex process that continuously adjusts to new conditions based on mechanical cues and cell–cell interactions. The collective results and forest plot (Figure 3) indicated a higher expression of a number of ECM degradation-related markers and lower expression of inhibitors of ECM degradation in SUI, but their relative contribution and role in disturbing the ECM metabolism cannot be judged. Based on this systematic review, there is insufficient evidence to conclude that these markers are the main contributors to the lower expression of ECM macromolecules.

A number of human [43,61] studies as well as an *Esr1* KO animal study [107] suggest an association of *ESR1* and *ESR2* with SUI emphasizing the importance of hormonal status. It is unclear if this has an effect on ECM metabolism, which should be further examined.

The clinical value of serum hormonal biomarkers in SUI patients remains unclear: the contribution of menopause and its accompanying hormonal changes in association with SUI could not be confirmed in this review. Clear initiators of this process, rather than a previous trauma (e.g., childbirth), prolonged pressure and aging, still need to be determined in order to devise new prophylactic or therapeutic strategies.

In addition to the aberrant ECM metabolism, increased expression of several pro-apoptotic, oxidative stress-related, and inflammation-related markers and the association of the Nfe2l2 gene in one animal knockout study [112] imply the association of an aberrant wound healing process in SUI. The lower expression of many neuronal cell-specific proteins and association with neurodegeneration-related proteins in SUI subjects is also in line with this and is possibly due to partly irreversible neurological damage after trauma or a neurodegenerative process involved in SUI. As for muscle cells, involvement of smooth muscle cell differentiation and/or contraction seems to be associated with SUI. The molecular mechanisms underlying these processes and their role in the pathogenesis of SUI should be further examined in depth.

The overarching theme appears to be an altered micromilieu and metabolism compared to controls. Which factors are most important for the development or continuation of SUI cannot be concluded. Furthermore, it should be further investigated how the important environmental risk factors that contribute to SUI—e.g., aging and trauma to the endopelvic fascia—impact on and interact with the identified (putative) molecular processes.

### 3.2. Comparison with Existing Literature

The findings of the current systematic review are in line with and expand the knowledge of previously published reviews [17,129,130,131,132]. The systematic review on gene expression differences of Isali et al., included studies until September 2017. They showed that 13 genes were differentially expressed in SUI and indicated the probable involvement of intermediate filament cytoskeleton organization and ECM organization in the pathogenesis of SUI [17]. The current systematic review adds a suggestive association of *ESR1* and *ESR2* with SUI, increased expression of pro-apoptotic, oxidative stress, and inflammation-related markers, lower protein expression of neuron-specific markers, and an association with a neurodegenerative process as well as impaired muscle cell differentiation and contractility, in addition to aberrant ECM metabolism being involved in SUI. Cartwright et al., performed a meta-analysis of rs1799750 of *MMP1* and rs1800013 of *COL1A1* with SUI and did not confirm a clear association [129]. They included two study abstracts we excluded based on the impossibility to assess the risk of bias, but this did not result in different findings. Campeau et al., examined the ECM and showed an association with SUI and suggested the involvement of sex hormones altering ECM metabolism [130]. Chen et al., examined ECM metabolism and reproductive hormones and showed a genetic predisposition to abnormal ECM remodeling, modulated by reproductive hormones, trauma, mechanical stress load and aging [131]. McKenzie et al., examined genetic influences on SUI and showed there was increasing evidence suggesting a genetic basis for the development of SUI, and that candidate genes related to ECM remodeling might be associated. They suggested that future research should focus on the influence of estrogen and progesterone on ECM proteins [132].

### 3.3. Strengths and Limitations

The strength of this review lies in the broadly examined area and inclusion of both human and animal studies. Despite the broadly examined area, the numbers of studies per selected outcome are still rather low. Moreover, the considerable heterogeneity between the included studies has prevented us from drawing firm conclusions based on the results of this systematic review, and below, we will discuss the main heterogeneity-related issues per study type.

Generally, in human studies many different definitions and methods for diagnosing SUI were used, ranging from self-reported to diagnosed via urodynamics. Subsequently, the definitions of the controls used were rather different, ranging from “continent controls” or “women without SUI” to women with other benign gynecological complaints or with cervical neoplasms. This variation in definition impacts negatively on the homogeneity of the studies. In addition, multiple different tissues/fluids were examined, e.g., ligaments, periurethral biopsies, vaginal wall, skin biopsies, urine, and serum. Studies often selected only one specific tissue, e.g., vaginal wall tissue in their assessment, which may only explain part of the SUI etiology or with unclear direct relevance in relation to SUI. Finally, some patients had comorbidities or used hormonal replacement therapy, and many studies did not mention (probable) medication use or comorbidities. It is likely that all this may have influenced the data by over or underestimating expression differences or genetic associations.

We included articles regardless of the co-existence with POP. When evaluating the data, we did not correct for the additional effect of POP on our outcomes. We concluded that excluding these studies would lead to introducing more (selection) bias because SUI so often co-exists with POP and because many studies did not mention the (possible) co-existence.

Thirty-five animal studies, using rats and mice, were included in this review. In particular, animal studies using specific gene knockout models appeared to be useful as they showed a direct causative association of certain genes with SUI. Although we believe that animal models can provide a valuable contribution to the knowledge of the pathophysiology of SUI, there are some pitfalls when evaluating the results: the studies generally lacked reporting of important topics of risk of bias, generally used young and healthy animals, and measured expression changes within a broad range after inducing SUI—of which the majority focused on a relatively short time frame post-induction. This contrasts starkly with SUI in humans where SUI usually develops in the years after childbirth [133]. This may explain conflicting results and questions whether translation to humans is feasible. Furthermore, it is well established that these rodents usually functionally recover within a relatively short period of time after induction of SUI and research on the long-term effects of SUI induction by various methods is scarce [134]. Therefore, for future research in animals related to gene and protein expression changes in SUI, especially these long-term effects should be considered in order to avoid the bias of examining a (normal) wound healing process instead of the actual development of SUI.

In addition to summarizing the data of studies without quantitative data, the forest plots were used to visualize differences in expression when comparing SUI subjects with controls. However, a pooled summary result of the calculated SMDs per examined gene/protein could not be determined, mainly because of the heterogeneity in the tissues examined, the low number of studies per examined gene/protein (as there was often data from only one study), and the different assay methods.

Very few studies included in this review were based on a hypothesis-free approach. Examining candidate genes and proteins is important and necessary in determining an exact contribution of a specific marker, but this kind of hypothesis-driven focus could lead to missing important contributors.

Of the human studies, only 57% scored low or medium low on the total judgment of risk of bias. Future studies should report on and match participants in age, parity, BMI, and possibly menopausal status and mode of delivery and should define and diagnose SUI in a valid and consistent manner. This will greatly improve the quality of the results. In terms of the risk of bias of animal studies, future studies using animal models to mimic SUI need to improve transparency and reporting standards.

## 4. Materials and Methods

### 4.1. Eligibility Criteria

This systematic review includes human and animal studies (domain) on genetic variants, gene and/or protein expression differences (outcome) in relation to SUI with or without POP (determinant) compared to controls. We used our prospectively registered protocol in Prospero (registration number CRD42019120202) on UI in general (Supplement S6) narrowing our selection process to SUI instead of UI in general. This beforementioned protocol was also used for our review on urgency urinary incontinence [135]. The inclusion criteria were studies with primary research data of affected cases (SUI) and controls of humans or animals (all species and sexes), examining genetic variants, gene expression, or protein expression differences, with sufficient information to determine the risk of bias.

### 4.2. Information Sources and Search Strategy

On 5 January 2021, a systematic search was performed in Pubmed, Embase, Web of Science, and the Cochrane library to identify available studies using the search strategy described in Supplement S7. The terms used were related to UI in general and a broad spectrum of genetic and protein expression terms and assays. References of reviews and included studies were cross-checked for studies not retrieved by the database search.

### 4.3. Study Selection

Studies were screened by two independent reviewers (WMP screened all articles, JW and HG shared the second screening) in two phases (title/abstract and full text phase) using the predefined inclusion criteria. In case only one of the reviewers assessed a study as eligible for full text screening, the study was screened full text by both reviewers. Potential discrepancies were discussed until consensus was reached, ultimately with the help of another author (KBK) when required. Studies with either solely SUI or both SUI and POP were included. Studies with SUI due to cancer-related surgical interventions were excluded from our review, due to possible confounding effects, e.g., of the malignancy.

### 4.4. Data Extraction

Extraction of study characteristics was performed by one and verified by a second reviewer. Characteristics extracted from the human studies were: first author, year of publication, number of cases/controls, definition and diagnostic-method of SUI, assessed material, assay method, investigated gene/protein/genetic variant, and results of the (value) differences between the groups (SUI and controls). From the animal studies, first author, year of publication, type of animal, sex, number of subjects, SUI induction method and confirmation of diagnosis, assessed material, assay method, investigated gene/protein/genetic variant, and results of the (value) differences between the groups (SUI and controls), were extracted. During data extraction, data of animals receiving treatment were not included due to possible confounding effects of this treatment on gene and protein expression. In addition, data with (naïve) cell-cultures being exposed to specific conditions (e.g., mechanical stress) without defining a clear SUI phenotype were not included. Only data of (induced) SUI versus controls was included. Therefore, for some studies in this review only part of their data was collected and reviewed. In the case of (animal) studies with measurements on multiple timepoints after induction of SUI, the data of the last timepoint was collected.

### 4.5. Assessment of Risk of Bias

The assessment process was carried out by one and checked by a second reviewer. Potential discrepancies were discussed until consensus was reached, ultimately with the help of another author when required. The Cochrane ROBINS-I [136] and SYRCLE risk of bias [137] tools were used for human and animal studies, respectively. In addition to the basic aspects of risk of bias assessment, the following topics were added: differences between cases and controls in age, BMI, menopausal status, parity, and mode of delivery. Similar to our review of urgency urinary incontinence [135], we added seven signaling questions to the risk of bias tools, addressing the risk of bias for in-vitro aspects of studies (Supplement S8), to be used when applicable. These questions were developed based on a tool for in vitro studies by the National Toxicology Program [138].

### 4.6. Data Synthesis

Due to the broad research topic of genetic variants, gene and protein expression changes in humans and animals related to SUI and including both quantitative and non-quantitative studies, it was not justifiable to estimate pooled effects using meta-analysis in this systematic review. The extracted data per outcome measure were grouped in themes, according to their functions in cellular processes. The genetic variants and changes in gene and protein expression were summarized within these themes. The results and differences between human and animal study outcomes were discussed, and differences per menopausal status were discussed when applicable. We did not make a distinction between menstrual phases. When applicable and in order to show probable trends in gene or protein expression differences, forest plots of (semi-)quantitative data showing standardized mean differences (SMDs) were drawn using the statistical software R version 4.1.0 [139] and package meta version 4.18-2 [140]. It was decided to present SMDs instead of mean differences because of the different ways of measuring the expression changes and different outcome measures.

For the forest plots, only quantitative or semi-quantitative data were taken into account. Semi-quantitative data (e.g., from scoring systems for staining) was converted into quantitative data in a standardized manner (by assigning the values 0/1/2 to −/+/++ or 1.1/1.2/1.3 to +/++/+++). Proportions were included, considering the proportion values as means. The standard deviations (SDs) were then calculated with the following formula SD = sqrt ((proportion) × (1-proportion)). If unclear whether SDs or standard errors (SEs) were shown, a conservative approach was taken assuming the provided numbers were SEs, and SDs were calculated using the following formula: SD = SE × sqrt (numbers of participants). If data needed to be combined, e.g., in case of studies distinguishing between menstrual phase (proliferative and secretory) using different participants, it was combined using the formulas from the Cochrane Handbook for Systematic Reviews of Interventions Version 6.2 [141].

Data presented in figures without providing the exact values and/or variability measures were excluded from the forest plots. When studies examined multiple tissues of one subject, the data of the most relevant tissue according to the authors of this review was included in the forest plot, in order to prevent multiplicity issues.

Publication bias was not evaluated because the data included were far too heterogeneous, and therefore, no meta-analysis could be performed. The certainty of the evidence was assessed using the Grading of Recommendations, Assessment, Development and Evaluation (GRADE) approach [142]. Given the nature of this systematic review, a modification was used, for which observational studies start with high certainty evidence.

## 5. Conclusions

This systematic review provides an overview of the current knowledge of genetic variants and gene and protein expression differences associated with SUI. The results suggest that altered ECM metabolism with a lower protein expression of ECM macromolecules and crosslinking compounds, estrogen receptors, oxidative stress, apoptosis, inflammation, neurodegenerative processes, and muscle cell differentiation and contractility seem to be associated with SUI.

Future research should focus on possible contributors to these alterations. The authors recommend the use of hypothesis-free approaches in order to find possible new links and candidate genes/proteins.

## Figures and Tables

**Table 1:** Human studies with hypothesis-free approach.

Author, Year	SUI/SUI + POP	Genome/Transcriptome/Proteome	Assay Methods	No. of Patients	No. of Controls	Summary of Findings
Penney et al., 2019	SUI	Genome	GWAS	1809	4811	No genome-wide significant SNPs, after adjusting for known risk factors, the top ranked SNP in the unadjusted SUI analysis became genome-wide significant (rs7607995, *p* = 4.5 × 10^−8^, chromosome 2p13.1, *WDR54*)
Chen et al., 2006	SUI	Transcriptome	Microarray, RT-PCR, WB, QC-PCR, immunofluorescence	17	19	Differential expression of 79 genes. Up-regulated genes (involved in ECM metabolism): skin-derived protease inhibitor 3 (elafin); IL-1RA; keratin 6, 14 and 16; and psoriasin 1. Downregulated genes: α2 actin; actin depolymerizing factor; smooth muscle myosin; light polypeptide kinase; RAMP-1; tropomyosin 1; microfibril-associated glycoprotein-2; insulinlike growth factor binding protein 7; collagen type IV α chain, several large cDNA genes (named KIAA).
Tong et al., 2010	SUI	Transcriptome	Microarray, RT-PCR, and IHC	9	8	Differential expression of 75 genes. The four most related pathways: solutable N-ethylmaleimidesensitive factor attachment protein receptor (SNARE) interactions in vesicular transport containing STX10, GOSR1 genes; neurodegenerative disorders containing GRB2, APOE genes; fructose and mannose metabolism containing TPl1, TSTA3 genes; and inositol metabolism containing GBA gene.
Liu et al., 2014	SUI	Transcriptome	Microarray, RT-PCR, WB	13	13	Differential expression of 12 miRNAs, three miRNA-mRNA pairs. Target genes are associated with neurodegenerative conditions
Wei et al., 2020	SUI	Transcriptome	Microarray, qRT-PCR	11	11	Differential expression of 8840 lncRNAs and 7102 mRNAs. Several lncRNAs are involved in the lysosome pathway associated with extracellular matrix (ECM) remodeling. Several mRNAs are involved in fibroblast pseudopodia formation, fibroblast growth, and the regulation of smooth muscle cell differentiation in the urinary tract.
Athanasiou et al., 2010	SUI+POP	Proteome	2-DE, MS and WB	4	3	Differential expression of seven proteins (more than two-fold): Overexpressed: Transgelin, Smooth muscle gamma-actin, myosin light polypeptide 6, precursor of alpha-1 antitrypsin, galectin-1. Underexpressed: two isoforms of transgelin. Only detected in patient group: type I keratin cytoskeletal 10 (CK10) and two isoforms of transgelin. These proteins are related to muscle contraction, cytoskeleton, cell maintenance, stability, and motility, smooth muscle differentiation, inhibition of extracellular matrix degradation, apoptosis.
Wen et al., 2012	SUI	Proteome	SELDI-TOF MS, IHC, WB, RT-PCR	10	10	Differential expression of SM-22a. Associated with Fibroblast-to-myofibroblast differentiation, wound healing.
Koch et al., 2016	SUI	Proteome	HPLCS and MS	20	20	828 proteins identified six significant differences. Higher in SUI: plasma serine protease inhibitor (SERPINA5), leucine-rich alpha-2-glycoprotein (LRG1), lysosomal alpha-glucosidase (GAA), and peptidyl-prolyl cis- trans isomerase A (PPIA), associated with inflammation, degradation of glycogen to glucose. Lower in SUI: uromodulin and TALPID3, associated with prevention of urinary tract infection, water/electrolyte balance, and kidney innate immunity, and ciliogenesis and sonic hedgehog/SHH signaling
Koch et al., 2018	SUI	Proteome	MS	19	19	7012 proteins identified, 33 proteins were detected in SUI, not in controls, involved in inflammatory response, response to cellular stress, coagulation and cytoskeleton stability/motility. Five proteins were detected in controls, not in SUI, involved in immune/DNA damage response.

Abbreviations: 2-DE Two-dimensional electrophoresis, ECM extracellular matrix, GWAS genome-wide association study, HPLCS High performance liquid chromatography separation, IHC immunohistochemistry, MS mass spectometry, PCR polymerase chain reaction, POP pelvic organ prolapse, SELDI TOF Surface-enhanced laser desorption/ionization time-of-flight, SNP single-nucleotide variant, SUI stress urinary incontinence, WB Western-blot.

**Table 2:** Animal studies with hypothesis-free approach.

Author, Year	Type of Animal, Strain, etc.	Genome Transcriptome Proteome	Assay Method	No. of SUI Subjects	No. of Controls	Summary of Findings
Lin et al., 2009	Sprague-Dawley rats, pregnant, primiparous	Transcriptome	Microarray	10	14	23 genes overexpressed and 19 genes underexpressed associated with: apoptosis, neuron related, Rho A/Rho kinase pathway related, smooth muscle related, TGF signaling pathway related, wnt/Frizzled signaling pathway related, cellular adhesion, cellular metabolism, and transcriptional regulation.
Chen et al., 2013	Virgin C57BL/6 strain mice, aged 6–8 weeks	Proteome	2D DIGE and LC–MS/MS, WB, immunofluorescence staining, and IHC	6	6	68 differentially expressed proteins, 19 proteins up-regulated and 49 were down-regulated. Involved in generation of precursor metabolites and energy, oxidation of reduction, regulation of apoptosis, and glycolysis. Myosin expression in the urethra was significantly decreased in the 8-mm VD group as compared with the non-instrumented control group
Chen et al., 2015	ACTB-Cre/*Esr1* knockout (*Esr1*−/−) mice and WT (*Esr1* +/+) mice	Proteome	Genotyping via PCR, IHC and WB	6x2	6	11 proteins differentially expressed in *Esr1*+/+ and *Esr1*−/− female mice. Five proteins were down-regulated (TPM3, DDAH2, DESM, TCTP, CAPR2) and six were up-regulated (MYL1, MLRS, MYL3, NDUS8, MYL1, UCHL1). Involved in muscle development, contraction, and regulation, as well as immune response (amphoterin signaling and phagocytosis), proteolysis, and cell adhesion (platelet aggregation and integrin-mediated cell–matrix adhesion).

NB Some of these studies also examine candidate genes, these results are not included in the table. Abbreviations: 2D DIGE Dimensional differential gel electrophoresis, IHC immunohistochemistry, LC–MS/MS liquid chromatography–tandem mass spectrometry, PCR Polymerase chain reaction, POP pelvic organ prolapse, SUI stress urinary incontinence, WB Western blot, WT wildtype.

**Table 3:** Certainty of Evidence assessment.

	Certainty Assessment						Certainty
	Study Design	Risk of Bias	Inconsistency	Indirectness	Imprecision	Publication Bias	
Extracellular matrix remodeling is associated with SUI	Observational studies	Serious ^1^	Not serious	Not serious	Serious ^4^	Not evaluated ^5^	Moderate
Estrogen receptor expression is associated with SUI in premenopausal women	Observational studies	Serious ^1^	Serious ^2^	Serious ^3^	Serious ^4^	Not evaluated ^5^	Low
Oxidative stress is associated with SUI	Observational studies	Serious ^1^	Serious ^2^	Serious ^3^	Serious ^4^	Not evaluated ^5^	Low
Apoptosis is associated with SUI	Observational studies	Serious ^1^	Serious ^2^	Serious ^3^	Serious ^4^	Not evaluated ^5^	Low
Inflammation is associated with SUI	Observational studies	Serious ^1^	Serious ^2^	Serious ^3^	Serious ^4^	Not evaluated ^5^	Low
Neurodegenerative processes are associated with SUI	Observational studies	Serious ^1^	Serious ^2^	Serious ^3^	Serious ^4^	Not evaluated ^5^	Low
Muscle cell differentiation is associated with SUI	Observational studies	Serious ^1^	Serious ^2^	Serious ^3^	Serious ^4^	Not evaluated ^5^	Low
Muscle cell contractility is associated with SUI	Observational studies	Serious ^1^	Serious ^2^	Serious ^3^	Serious ^4^	Not evaluated ^5^	Low

Explanation: ^1^ Downgraded one level for risk of bias; ^2^ Downgraded one lever for inconsistency (heterogeneity in study outcomes); ^3^ Downgraded one level for indirectness (use of animal models); ^4^ Downgraded one level for imprecision (relatively low number of studies per outcome); ^5^ This was not evaluated due to low number of studies per marker. Abbreviations: SUI stress urinary incontinence.

## Data Availability

No new data were created or analyzed in this study. Data sharing is not applicable to this article.

## Supplementary Materials

The following supporting information can be downloaded at: www.mdpi.com/article/10.3390/ijms23063401/s1.
