# Supplementary material for: Molecular Processes in Stress Urinary Incontinence: A Systematic Review of Human and Animal Studies
Source: Int J Mol Sci. 2022 Mar 21;23(6):3401. doi: 10.3390/ijms23063401 (PMC8949972; doi:10.3390/ijms23063401)
Supplement: Supplementary file 1 [file ijms-23-03401-s001.zip › Supplement 6 Review protocol Prospero.pdf]

National Institute for Health Research

University of York
Centre for Reviews and Dissemination

Systematic review

1. * Review title.

Give the working title of the review, for example the one used for obtaining funding. Ideally the title should state succinctly the interventions or exposures being reviewed and the associated health or social problems. Where appropriate, the title should use the PI(E)COS structure to contain information on the Participants, Intervention (or Exposure) and Comparison groups, the Outcomes to be measured and Study designs to be included.

Genetic variations and expression changes in urinary incontinence: a systematic review

2. Original language title.

For reviews in languages other than English, this field should be used to enter the title in the language of the review. This will be displayed together with the English language title.

3. * Anticipated or actual start date.

Give the date when the systematic review commenced, or is expected to commence.
09/10/2018

4. * Anticipated completion date.

Give the date by which the review is expected to be completed.
01/08/2019

5. * Stage of review at time of this submission.

Indicate the stage of progress of the review by ticking the relevant Started and Completed boxes. Additional information may be added in the free text box provided.
Please note: Reviews that have progressed beyond the point of completing data extraction at the time of initial registration are not eligible for inclusion in PROSPERO. Should evidence of incorrect status and/or completion date being supplied at the time of submission come to light, the content of the PROSPERO record will be removed leaving only the title and named contact details and a statement that inaccuracies in the stage of the review date had been identified.
This field should be updated when any amendments are made to a published record and on completion and publication of the review. If this field was pre-populated from the initial screening questions then you are not able to edit it until the record is published.

The review has not yet started: No

Review stage	Started	Completed
Preliminary searches	Yes	Yes
Piloting of the study selection process	Yes	No
Formal screening of search results against eligibility criteria	Yes	No
Data extraction	No	No
Risk of bias (quality) assessment	No	No
Data analysis	No	No

Provide any other relevant information about the stage of the review here (e.g. Funded proposal, protocol not yet finalised).

6. * Named contact.

The named contact acts as the guarantor for the accuracy of the information presented in the register record.

Wilke Post

Email salutation (e.g. "Dr Smith" or "Joanne") for correspondence:

Wilke Post

7. * Named contact email.

Give the electronic mail address of the named contact.

wilke.post@radboudumc.nl

8. Named contact address

Give the full postal address for the named contact.

drs. Wilke Post (791 GYN), Postbus 9101, 6500 HB Nijmegen

9. Named contact phone number.

Give the telephone number for the named contact, including international dialling code.

10. * Organisational affiliation of the review.

Full title of the organisational affiliations for this review and website address if available. This field may be completed as 'None' if the review is not affiliated to any organisation.

Radboudumc, Nijmegen, the Netherlands

Organisation web address:

11. * Review team members and their organisational affiliations.

Give the title, first name, last name and the organisational affiliations of each member of the review team. Affiliation refers to groups or organisations to which review team members belong.

Miss Wilke Post. Radboudumc
Dr Egbert Oosterwijk. Radboud university medical center, Department of Urology, Nijmegen, The Netherlands.

Miss Hilde Grens. Radboud university medical center, Department of Gynaecology, Nijmegen, The Netherlands.
Dr Rob B.M. de Vries. Radboud university medical center, Department for Health Evidence, Nijmegen, The Netherlands.
Dr Kirsten B. Kluivers. Radboud university medical center, Department of Gynaecology, Nijmegen, The Netherlands.

12. * Funding sources/sponsors.

Give details of the individuals, organizations, groups or other legal entities who take responsibility for initiating, managing, sponsoring and/or financing the review. Include any unique identification numbers assigned to the review by the individuals or bodies listed.

EFRO (European Fund of regional developments), ZonMw, Radboudumc

13. * Conflicts of interest.

List any conditions that could lead to actual or perceived undue influence on judgements concerning the main topic investigated in the review.

None

14. Collaborators.

Give the name and affiliation of any individuals or organisations who are working on the review but who are not listed as review team members.

15. * Review question.

State the question(s) to be addressed by the review, clearly and precisely. Review questions may be specific or broad. It may be appropriate to break very broad questions down into a series of related more specific questions. Questions may be framed or refined using PI(E)COS where relevant.

Which genetical variations, gene- expression, or protein expression changes are associated with urinary incontinence in humans and animals and in vitro studies?

16. * Searches.

State the sources that will be searched. Give the search dates, and any restrictions (e.g. language or publication period). Do NOT enter the full search strategy (it may be provided as a link or attachment.)

PubMed, Embase, Web of Science, Cochrane. 9th October 2018 the search was performed. No restrictions.

Additional search strategy information can be found in the attached PDF document (link provided below).

17. URL to search strategy.

Give a link to a published pdf/word document detailing either the search strategy or an example of a search strategy for a specific database if available (including the keywords that will be used in the search strategies), or upload your search strategy.Do NOT provide links to your search results.

https://www.crd.york.ac.uk/PROSPEROFILES/120202_STRATEGY_20190611.pdf

Alternatively, upload your search strategy to CRD in pdf format. Please note that by doing so you are consenting to the file being made publicly accessible.

Do not make this file publicly available until the review is complete

18. * Condition or domain being studied.

Give a short description of the disease, condition or healthcare domain being studied. This could include

health and wellbeing outcomes.

We study genetic variations and expression changes (both gene and proteinexpression) in the development and existing stage of urinary incontinence (urgency, stress and mixed) in both humans and (induced) in animals.

The domain is (cells/tissues of) humans and animals.

19. * Participants/population.

Give summary criteria for the participants or populations being studied by the review. The preferred format includes details of both inclusion and exclusion criteria.

Inclusion: (tissue/cells of) humans/animals, male/female, of all ages, living/deceased, no comorbidities.

Exclusion: comorbidities.

20. * Intervention(s), exposure(s).

Give full and clear descriptions or definitions of the nature of the interventions or the exposures to be reviewed.

Inclusion: presence of (idiopathic) stress-, urgency or mixed urinary incontinence, defined as involuntary loss of urine spontaneously or induced (e.g. by increased abdominal pressure), reduced leak point pressure, or diagnosed detrusor overactivity in combination with involuntary loss of urine, (OR in case of animal/in vitro studies: other defined urinary incontinence model).

21. * Comparator(s)/control.

Where relevant, give details of the alternatives against which the main subject/topic of the review will be compared (e.g. another intervention or a non-exposed control group). The preferred format includes details of both inclusion and exclusion criteria.

No urinary incontinence.

22. * Types of study to be included.

Give details of the types of study (study designs) eligible for inclusion in the review. If there are no restrictions on the types of study design eligible for inclusion, or certain study types are excluded, this should be stated. The preferred format includes details of both inclusion and exclusion criteria.

Type of study (design):

Inclusion: Primary research data, affected cases and (own) controls. Article/ paper contains sufficient information to determine risk of bias. Clinical studies, animal studies and in vitro studies.

Exclusion: No primary research data, no controls included, no sufficient information to determine risk of bias.

Type of populations:

Inclusion: (tissue/cells of) humans/animals, male/female of all ages, no comorbidities.

Exclusion: comorbidities.Type of 'intervention' (determinant): Inclusion: Presence of (idiopathic) stress-, urgency or mixed urinary incontinence, defined as involuntary loss of urine spontaneously or induced (e.g. by increased abdominal pressure), reduced leak point pressure, or diagnosed detrusor overactivity in

combination with involuntary loss of urine, (or in case of animal/in vitro studies: other defined urinary incontinence model). And (matched) control group.

Outcome measures:

Inclusion: genetic variations, gene expression, or protein expression analysis of endogenous genes.

Exclusion: therapy related gene or protein expression changes, not gene or protein expression or genetic variations.

Language:

Inclusion: English, Dutch.

Exclusion: Chinese, Russian, Japanese, Korean.

23. Context.

Give summary details of the setting and other relevant characteristics which help define the inclusion or exclusion criteria.

24. * Main outcome(s).

Give the pre-specified main (most important) outcomes of the review, including details of how the outcome is defined and measured and when these measurement are made, if these are part of the review inclusion criteria.

Genetic variations (e.g. SNP's, mutations), gene expression levels (fold induction or qPCR cycle difference), or protein expression differences (e.g. differences in staining, fold changes) in urinary incontinence compared to subjects without the trait.

Timing and effect measures

Not applicable.

25. * Additional outcome(s).

List the pre-specified additional outcomes of the review, with a similar level of detail to that required for main outcomes. Where there are no additional outcomes please state 'None' or 'Not applicable' as appropriate to the review

None.

Timing and effect measures

Not applicable.

26. * Data extraction (selection and coding).

Describe how studies will be selected for inclusion. State what data will be extracted or obtained. State how this will be done and recorded.

Screening/selection:All screening phases will be performed by 2 reviewers independently. In the pre-screening phase, papers will be included if they are included by one of the reviewers, discrepancies in the

full-text screening phase will be discussed until an agreement has been reached. The remaining articles will be discussed with a third opinion.

Extracted data:

1 reviewer will extract the data from the included studies and random checks will be performed by a second reviewer.

27. * Risk of bias (quality) assessment.

Describe the method of assessing risk of bias or quality assessment. State which characteristics of the studies will be assessed and any formal risk of bias tools that will be used.

Two reviewers will assess the risk of bias, a third reviewer will be added in case of discrepancies.

28. * Strategy for data synthesis.

Provide details of the planned synthesis including a rationale for the methods selected. This **must not be generic text** but should be **specific to your review** and describe how the proposed analysis will be applied to your data.

It is anticipated that the studies identified in this review will be diverse (study design, study quality, methods described, different definitions of UI, different outcome measures) and therefore it is expected that a narrative synthesis will be performed. Text and tables will be used to provide a descriptive summary and explanation of study characteristics and findings.

29. * Analysis of subgroups or subsets.

State any planned investigation of ‘subgroups’. Be clear and specific about which type of study or participant will be included in each group or covariate investigated. State the planned analytic approach.

None planned.

30. * Type and method of review.

Select the type of review and the review method from the lists below. Select the health area(s) of interest for your review.

Type of review

Cost effectiveness
No

Diagnostic
Yes

Epidemiologic
No

Individual patient data (IPD) meta-analysis
No

Intervention
No

Meta-analysis
No

Methodology
No

Narrative synthesis

Yes

Network meta-analysis
No

Pre-clinical
No

Prevention
No

Prognostic
No

Prospective meta-analysis (PMA)
No

Review of reviews
No

Service delivery
No

Synthesis of qualitative studies
No

Systematic review
Yes

Other
No

Health area of the review

Alcohol/substance misuse/abuse
No

Blood and immune system
No

Cancer
No

Cardiovascular
No

Care of the elderly
No

Child health
No

Complementary therapies
No

Crime and justice
No

Dental
No

Digestive system
No

Ear, nose and throat
No

Education
No

Endocrine and metabolic disorders
No

Eye disorders
No

General interest
No

Genetics

No
Health inequalities/health equity
No
Infections and infestations
No
International development
No
Mental health and behavioural conditions
No
Musculoskeletal
No
Neurological
No
Nursing
No
Obstetrics and gynaecology
Yes
Oral health
No
Palliative care
No
Perioperative care
No
Physiotherapy
No
Pregnancy and childbirth
No
Public health (including social determinants of health)
No
Rehabilitation
No
Respiratory disorders
No
Service delivery
No
Skin disorders
No
Social care
No
Surgery
No
Tropical Medicine
No
Urological
Yes
Wounds, injuries and accidents
No
Violence and abuse
No

31. Language.

Select each language individually to add it to the list below, use the bin icon to remove any added in error.
English

There is not an English language summary

32. * Country.

Select the country in which the review is being carried out from the drop down list. For multi-national collaborations select all the countries involved.

Netherlands

33. Other registration details.

Give the name of any organisation where the systematic review title or protocol is registered (such as with The Campbell Collaboration, or The Joanna Briggs Institute) together with any unique identification number assigned. (N.B. Registration details for Cochrane protocols will be automatically entered). If extracted data will be stored and made available through a repository such as the Systematic Review Data Repository (SRDR), details and a link should be included here. If none, leave blank.

34. Reference and/or URL for published protocol.

Give the citation and link for the published protocol, if there is one

Give the link to the published protocol.

Alternatively, upload your published protocol to CRD in pdf format. Please note that by doing so you are consenting to the file being made publicly accessible.

No I do not make this file publicly available until the review is complete

Please note that the information required in the PROSPERO registration form must be completed in full even if access to a protocol is given.

35. Dissemination plans.

Give brief details of plans for communicating essential messages from the review to the appropriate audiences.

Do you intend to publish the review on completion?

Yes

36. Keywords.

Give words or phrases that best describe the review. Separate keywords with a semicolon or new line. Keywords will help users find the review in the Register (the words do not appear in the public record but are included in searches). Be as specific and precise as possible. Avoid acronyms and abbreviations unless these are in wide use.

Urinary incontinence; expression changes; genetic variations

37. Details of any existing review of the same topic by the same authors.

Give details of earlier versions of the systematic review if an update of an existing review is being registered, including full bibliographic reference if possible.

38. * Current review status.

Review status should be updated when the review is completed and when it is published. For newregistrations the review must be Ongoing.
Please provide anticipated publication date

Review_Ongoing

39. Any additional information.

Provide any other information the review team feel is relevant to the registration of the review.

Cartwright R, Kirby AC, Tikkinen KAO, et al. Systematic review and metaanalysis of genetic association studies of urinary symptoms and prolapse in women. Am J Obstet Gynecol 2015;212:199.e1-24.

This review perfomed by Cartwright et al. was performed on genetic association studies and did not focus on urinary incontinence solely. Thereby this review is not about expression changes.

40. Details of final report/publication(s).

This field should be left empty until details of the completed review are available.

Give the link to the published review.
