# Supplementary material for: Molecular Processes in Stress Urinary Incontinence: A Systematic Review of Human and Animal Studies
Source: Int J Mol Sci. 2022 Mar 21;23(6):3401. doi: 10.3390/ijms23063401 (PMC8949972; doi:10.3390/ijms23063401)
Supplement: Supplementary file 1 [file ijms-23-03401-s001.zip › Supplement 7 - Search 5-1-21.pdf]

# SR: Genetic variations, gene-and protein expression changes in urinary incontinence

*Search conducted on 05-01-2021*

## Pubmed

**Part 1:** (("Lower Urinary Tract Symptoms"[Mesh] OR "Urinary Bladder, Overactive"[Mesh] OR "Urinary Incontinence"[Mesh] OR "Enuresis"[Mesh] OR "Nocturia"[Mesh])) OR (Bladder incontinence[tiab] OR bladder overactivity[tiab] OR detrusor overactivity[tiab] OR Enuresis[tiab] OR involuntary void*[tiab] OR Nocturia[tiab] OR Nycturia[tiab] OR overactive bladder[tiab] OR overactive detrusor[tiab] OR Overactive Urinary Bladder[tiab] OR "overactivity of the bladder"[tiab] OR "overactivity of the detrusor"[tiab] OR "Overactivity of the Urinary Bladder"[tiab] OR Stress incontinence[tiab] OR Urge incontinence[tiab] OR Urinary Bladder Overactivity [tiab] OR Urinary incontinence[tiab] OR urinary loss[tiab] OR "Urinary Reflex Incontinence"[tiab] OR Urine incontinence[tiab] OR Urine loss[tiab] OR urine wetting[tiab] OR (urgency[tiab] AND (urinary[tiab] OR urine[tiab])))

**Part 2:** "genetics" [Subheading] OR "Genetics"[Mesh] OR Models, Molecular[MeSH] OR Genetic Techniques[meSH] OR Gene Expression Regulation[MeSH] OR Genotype[MeSH] OR "Genetic Variation"[Mesh] OR Gene expression[MeSH] OR Microarray Analysis[MeSH] OR Proteome[MeSH] OR immunoassay[MeSH] OR histology[MeSH] OR "Immunohistochemistry"[Mesh] OR "Staining and Labeling"[Mesh:NoExp] OR
enzyme[tiab] OR enzymes[tiab] OR epigenetic*[tiab] OR expression[tiab] OR gene[tiab] OR genes[tiab] OR genetic[tiab] OR genetics[tiab] OR genome[tiab] OR genome-wide[tiab] OR Genotyp*[tiab] OR genotype-phenotype[tiab] OR GWA stud*[tiab] OR GWAS[tiab] OR heredity[tiab] OR hereditary familial[tiab] OR inherited[tiab] OR microarray*[tiab] OR mRNA[tiab] OR mutation*[tiab] OR phenotype*[tiab] OR polymorfism[tiab] OR polymorfisms[tiab] OR Polymorphism[tiab] OR Polymorphisms[tiab] OR protein[tiab] OR proteins[tiab] OR Proteogenomic*[tiab] OR Proteome[tiab] OR proteomic*[tiab] OR sequence analysis[tiab] OR sequencing[tiab] OR SNP[tiab] OR transcriptome[tiab] OR transcriptomic[tiab] OR immunohistochemistry[tiab] OR elisa[tiab] OR (western[tiab] AND (blot[tiab] OR blots[tiab] OR blotting[tiab])) OR histology[tiab] OR PCR[tiab] OR Immunocytochemistry[tiab]

**Hits: 3185**

## Embase

**Part 1:**

exp urine incontinence/ or overactive bladder/ or (Bladder incontinence or bladder overactivity or detrusor overactivity or Enuresis or involuntary void* or Nocturia or Nycturia or overactive bladder or overactive detrusor or Overactive Urinary Bladder or "overactivity of the bladder" or "overactivity of the detrusor" or "Overactivity of the Urinary Bladder" or Stress incontinence or Urge incontinence or Urinary Bladder Overactivity or Urinary incontinence or urinary loss or "Urinary Reflex Incontinence" or Urine incontinence or Urine loss or Urine wetting or (urgency and (urinary or urine))).ti,ab,kw.

**Part 2:**

(enzyme or enzymes or epigenetic* or expression or gene or genes or genetic or genetics or genome or genome-wide or Genotyp* or genotype-phenotype or GWA stud* or GWAS or heredity or hereditary familial or inherited or microarray* or mRNA or mutation* or phenotype* or polymorfism or polymorfisms or Polymorphism or Polymorphisms or protein or proteins or Proteogenomic* or Proteome or proteomic* or sequence analysis or sequencing or SNP or transcriptome or transcriptomic* or immunohistochemistry OR elisa OR (western AND (blot OR blots OR blotting)) OR histology OR PCR OR Immunocytochemistry).ti,ab,kw. Or exp genetics/ or exp proteomics/ or transcriptomics/ or Molecular model/ or exp genetic procedures/ or exp genetic variation/ or exp allelism/ or genetic association/ or genetic background/ or exp genetic compatibility/ or genetic complementation/ or genetic correlation/ or exp genetic cross/ or exp genetic damage/ or exp genetic heterogeneity/ or exp genetic line/ or genetic organization/ or exp genetic resource/ or genetic trait/ or exp genome/ or exp genotype/ or heritability/ or exp inheritance/ or microsatellite instability/ or exp mutation/ or exp organism hybridization/ or exp phenotype/ or exp genetic difference/ or exp genetic linkage/ or exp genetic marker/ or exp genetic polymorphism/ or genetic variability/ or genotype phenotype correlation/ or exp enzyme activity/ or exp enzyme chemistry/ or exp enzyme inhibition/ or exp enzyme kinetics/ or exp enzyme metabolism/ or exp enzyme polymorphism/ or exp enzyme regulation/ or exp enzyme structure/ or exp gene activity/ or exp gene expression/ or exp gene mutation/ or exp genetic polymorphism/ or exp genetic regulation/ or exp genetic variation/ or microarray analysis/ or protein microarray/ or proteome/ or histology/ or histochemistry/ or immunohistology/ OR immunohistochemistry/ OR immunochemistry/ or exp fluorescent antibody technique/ or exp immunoassay/ or immunocytochemistry/ OR western blotting/ OR staining/ OR polymerase chain reaction/ or real time polymerase chain reaction/ or reverse transcription polymerase chain reaction/

**Hits: 8689**

**Web of Science**

**Part 1:**

“Bladder incontinence” OR “bladder overactivity” OR “detrusor overactivity” OR Enuresis OR “involuntary void*” OR Nocturia OR Nycturia OR “overactive bladder” OR “overactive detrusor” OR “Overactive Urinary Bladder” OR "overactivity of the bladder" OR "overactivity of the detrusor" OR "Overactivity of the Urinary Bladder" OR “Stress incontinence” OR “Urge incontinence” OR “Urinary Bladder Overactivity” OR “Urinary incontinence” OR “urinary loss” OR "Urinary Reflex Incontinence" OR “Urine incontinence” OR “Urine loss” OR “urine wetting” OR (urgency AND (urinary OR urine))

**Part 2:**

(enzyme or enzymes or epigenetic* or expression or gene or genes or genetic or genetics or genome or genome-wide or Genotyp* or genotype-phenotype or “GWA stud*” or GWAS or heredity or hereditary or familial or inherited or microarray* or mRNA or mutation* or phenotype* or polymorfism or polymorfisms or Polymorphism or Polymorphisms or protein or proteins or Proteogenomic* or Proteome or proteomic* or “sequence analysis” or sequencing or SNP or transcriptome or transcriptomic*or immunohistochemistry OR elisa OR (western AND (blot OR blots OR blotting)) OR histology OR PCR OR Immunocytochemistry)

**Hits: 3752**

## Cochrane

**Part 1:**

“Bladder incontinence” OR “bladder overactivity” OR “detrusor overactivity” OR Enuresis OR “involuntary void*” OR Nocturia OR Nycturia OR “overactive bladder” OR “overactive detrusor” OR “Overactive Urinary Bladder” OR "overactivity of the bladder" OR "overactivity of the detrusor" OR "Overactivity of the Urinary Bladder" OR “Stress incontinence” OR “Urge incontinence” OR “Urinary Bladder Overactivity” OR “Urinary incontinence” OR “urinary loss” OR "Urinary Reflex Incontinence" OR “Urine incontinence” OR “Urine loss” OR “urine wetting” OR (urgency AND (urinary OR urine))

**Part 2:**

enzyme or enzymes or epigenetic* or expression or gene or genes or genetic or genetics or genome or genome-wide or Genotyp* or genotype-phenotype or “GWA stud*” or GWAS or heredity or hereditary or familial or inherited or microarray* or mRNA or mutation* or phenotype* or polymorfism or polymorfisms or Polymorphism or Polymorphisms or protein or proteins or Proteogenomic* or Proteome or proteomic* or “sequence analysis” or sequencing or SNP or transcriptome or transcriptomic*or immunohistochemistry OR elisa OR (western AND (blot OR blots OR blotting)) OR histology OR PCR OR Immunocytochemistry

**Hits: 720**
