# Supplementary material for: Molecular Processes in Stress Urinary Incontinence: A Systematic Review of Human and Animal Studies
Source: Int J Mol Sci. 2022 Mar 21;23(6):3401. doi: 10.3390/ijms23063401 (PMC8949972; doi:10.3390/ijms23063401)
Supplement: Supplementary file 1 [file ijms-23-03401-s001.zip › Supplement 8 in vitro questions.pdf]

In-vitro questions

3. Were experimental conditions identical across study groups?

Definitely Low Risk of Bias (++)

- Direct evidence that culture conditions included identical concentrations of any solvents (e.g., DMSO) used in getting the treatment compound into solution,
- **AND** the same media was used for control and experimental cells particularly for biological materials such as serum which must be from the same lot,
- **AND** appropriate adjustments were made such as normalization to blank/media controls, cell numbers in culture, use of positive and negative control responses in acceptance criteria, or others,
- **AND** non-treatment-related experimental conditions were identical across study groups (i.e., the study report explicitly provides this level of detail).

Probably Low Risk of Bias (+)

- Indirect evidence that culture conditions included identical concentrations of any solvents (e.g., DMSO) used in getting the treatment compound into solution,
- **AND** the same media was used for control and experimental cells,
- **OR** it is deemed that the media used would not appreciably bias results,
- **AND** appropriate adjustments were made such as normalization to blank/media controls, cell numbers in culture, use of positive and negative control responses in acceptance criteria, or others,
- **OR** it is deemed that not considering or only considering a partial list of covariates in the final analyses would not appreciably bias results,
- **AND** as described above, identical non-treatment-related experimental conditions are assumed if authors did not report differences in culture conditions or handling.

Probably High Risk of Bias (-) or (NR)

- Indirect evidence that the concentration of solvents used in getting the treatment compound into solution differed between control and experimental cells,
- **OR** there is indirect evidence that the media differed between control and experimental cells,
- **OR** there is insufficient information provided on maintaining identical concentrations of solvents (record “NR” as basis for answer),
- **OR** there is indirect evidence that appropriate adjustments were not made such as failing to normalize to blank/media controls, adjust for cell numbers in culture, use positive and negative control responses in acceptance criteria, or others,
- **OR** there is insufficient information provided about analysis of relevant covariates (record “NR” as basis for answer),
- **OR** there is indirect evidence that non-treatment-related experimental conditions were not comparable between study groups.

Definitely High Risk of Bias (--)

- Direct evidence from the study report that the concentration of solvents used in getting the treatment compound into solution differed between control and experimental cells,
- **OR** there is direct evidence that the media (or biological components such as serum) differed between control and experimental cells,
- **OR** there is direct evidence that appropriate adjustments were not made such as failing to normalize to blank/media controls, adjust for cell numbers in culture, use positive and negative control responses in acceptance criteria, or other relevant covariates,
- **OR** there is direct evidence that non-treatment-related experimental conditions were not comparable between study groups.

4. Were the research personnel blinded to the study group during the study?

Definitely Low Risk of Bias (++)
• Direct evidence that the research personnel were adequately blinded to study group, and it is unlikely that they could have broken the blinding during the study. Methods used to ensure blinding include central allocation, sequentially numbered treatment containers of identical appearance; sequentially numbered culture plates, or equivalent,<br>• **OR** the use of robotic testing systems during the study that are deemed to eliminate the opportunity for performance bias to influence results.
**Probably Low Risk of Bias (+)**
• Indirect evidence that the research personnel were adequately blinded to study group, and it is unlikely that they could have broken the blinding during the study,<br>• **OR** it is deemed that lack of adequate blinding during the study would not appreciably bias results (e.g., minimal possibility of researchers to handle cells or plates after treatment due to primarily automated procedures).
**Probably High**
• Indirect evidence that the research personnel were not adequately blinded to study group,<br>• **OR** there is insufficient information provided about blinding to study group during the study (record "NR" as basis for answer).
**Definitely High Risk of Bias (--)**
• Direct evidence that the research personnel were not adequately blinded to study group.

5. Were outcome data complete without attrition or exclusion from analysis?

Definitely Low Risk of Bias (++)
• Direct evidence that loss of cells was adequately addressed and reasons were documented when wells or plates were removed from a study (e.g., visual observation of contamination, cells missing from wells due to pipetting error, visual morphological changes in cells unexplainable based on surrounding wells, documented removal of statistical outliers).<br>• **Note**: Acceptable handling of attrition includes: very little missing outcome data; reasons for missing cells unlikely to be related to outcome (or for viability data, censoring unlikely to be introducing bias); missing outcome data balanced in numbers across study groups, with similar reasons for missing data across groups; missing outcomes is not enough to impact the effect.
**Probably Low Risk of Bias (+)**
• Indirect evidence that loss of cells was adequately addressed and reasons were documented when wells or plates were removed from a study,<br>• **OR** it is deemed that the proportion lost would not appreciably bias results.
**Probably High Risk of Bias (-) or (NR)**
• Indirect evidence that loss of wells or culture plates was unacceptably large and not adequately addressed,<br>• **OR** there is insufficient information provided about loss of cells (record "NR" as basis for answer).

Definitely High Risk of Bias (--)
• Direct evidence that loss of cells, wells, or plates was unacceptably large and not adequately addressed.<br>• **Note**: Unacceptable handling of attrition or exclusion includes: reason for loss is likely to be related to true outcome, with either imbalance in numbers or reasons for loss across study groups.

6. Can we be confident in the outcome assessment?

Definitely Low Risk of Bias (++)
• Direct evidence that the outcome was assessed using well-established methods (the gold standard),<br>• **AND** assessed at the same length of time after initial exposure in all study groups,<br>• **AND** there is direct evidence that the outcome assessors were adequately blinded to the study group, and it is unlikely that they could have broken the blinding prior to reporting outcomes.
• **NOTE** Well-established methods will depend on the outcome, but examples of such methods may include: objectively measured cytokine concentrations with diagnostic methods using commercial kits, commercial laboratories with experience in the assay, or standard assays such as ELISAs for IgG and with sufficiently low variation and limits of detection to allow discrimination of responses between treatment groups (or direct evidence that the assay could have detected a difference based on responses to a positive control).
**Probably Low Risk of Bias (+)**
• Indirect evidence that the outcome was assessed using acceptable methods (i.e., deemed valid and reliable but not the gold standard),<br>• **AND** assessed at the same length of time after initial exposure in all study groups,<br>• **OR** it is deemed that the outcome assessment methods used would not appreciably bias results,<br>• **AND** there is indirect evidence that the outcome assessors were adequately blinded to the study group, and it is unlikely that they could have broken the blinding prior to reporting outcomes,<br>• **OR** it is deemed that lack of adequate blinding of outcome assessors would not appreciably bias results, which is more likely to apply to objective outcome measures.<br>• **NOTE** Acceptable assessment methods will depend on the outcome, but examples of such methods may include: objectively measured antibody or cytokine concentrations with diagnostic methods using commercial kits with some variation, but ability to discriminate between the high dose treatment and control group (or indirect evidence that the assay could have detected a difference based on responses to a positive control).
**Probably High Risk of Bias (-) or (NR)**
• Indirect evidence that the outcome assessment method is an insensitive instrument,<br>• **OR** the length of time after initial exposure differed by study group,<br>• **OR** there is indirect evidence that it was possible for outcome assessors to infer the study group prior to reporting outcomes without sufficient quality control measures,<br>• **OR** there is insufficient information provided about blinding of outcome assessors (record "NR" as basis for answer).
**Definitely High Risk of Bias (--)**
• Direct evidence that the outcome assessment method is an insensitive instrument,<br>• **OR** the length of time after initial exposure differed by study group,<br>• **OR** there is direct evidence for lack of adequate blinding of outcome assessors, including no blinding or incomplete blinding without quality control measures.

7. Were all measured outcomes reported?

Definitely Low Risk of Bias (++)
• Direct evidence that all of the study's measured outcomes (primary and secondary) outlined in the protocol, methods, abstract, and/or introduction (that are relevant for the evaluation) have been reported. This would include outcomes reported with sufficient detail to be included in meta-analysis or fully tabulated during data extraction and analyses had been planned in advance.
**Probably Low Risk of Bias (+)**

- Indirect evidence that all of the study's measured outcomes (primary and secondary) outlined in the protocol, methods, abstract, and/or introduction (that are relevant for the evaluation) have been reported,
- **OR** analyses that had not been planned in advance (i.e., retrospective unplanned subgroup analyses) are clearly indicated as such and deemed that unplanned analyses were appropriate and selective reporting would not appreciably bias results (e.g., appropriate analyses of an unexpected effect). This would include outcomes reported with insufficient detail such as only reporting that results were statistically significant (or not).

Probably High Risk of Bias (-) or (NR)

- Indirect evidence that all of the study's measured outcomes (primary and secondary) outlined in the protocol, methods, abstract, and/or introduction (that are relevant for the evaluation) have not been reported,
- **OR** and there is indirect evidence that unplanned analyses were included that may appreciably bias results,
- **OR** there is insufficient information provided about selective outcome reporting (record "NR" as basis for answer).

Definitely High Risk of Bias (--)

- Direct evidence that all of the study's measured outcomes (primary and secondary) outlined in the protocol, methods, abstract, and/or introduction (that are relevant for the evaluation) have not been reported. In addition to not reporting outcomes, this would include reporting outcomes based on composite score without individual outcome components or outcomes reported using measurements, analysis methods or subsets of the data (e.g., subscales) that were not pre-specified or reporting outcomes not pre-specified, or that unplanned analyses were included that would appreciably bias results.

8. Can we be confident in the outcome of assays?

Definitely Low Risk of Bias (++)

- Direct evidence that all of the assays were performed with includement of 'control' genes/protein assays (i.e. genes/proteins which are and/or are not likely to be affected by the experiment).
- AND Direct evidence that all of the assays were performed in the same analysers (per gene)

Probably Low Risk of Bias (+)

- Indirect evidence that all of the assays were performed with includement of 'control' genes/protein assays (i.e. genes/proteins which are and/or are not likely to be affected by the experiment.

OR analyses that had not been planned in advance (i.e., retrospective unplanned subgroup analyses) are clearly indicated as such and deemed that unplanned analyses were appropriate and selective reporting would not appreciably bias results (e.g., appropriate analyses of an unexpected effect). This would include outcomes reported with insufficient detail such as only reporting that results were statistically significant (or not). **AND/OR** Indirect evidence that all of the assays were performed in the same analysers (per gene)

Probably High Risk of Bias (-) or (NR)

- (In)direct evidence that all of the assays were performed without includement of 'control' genes/protein assays (i.e. genes/proteins which are and/or are not likely to be affected by the experiment.
- **AND** (In)direct evidence that not all of the assays were performed in the same analysers (per gene)

9.Were there no other potential threats to internal validity?

There are no urinary incontinence specific additions to the risk-of-bias questions for this evaluation. This question will be used to examine individual studies for appropriate statistical methods (e.g., confirmation of homogeneity of variance for ANOVA and other statistical tests that require normally distributed data). It will also be used for risk-of-bias considerations that do not fit under the other questions.
